# Higher platelet count, even within normal range, is associated with increased arterial stiffness in young and middle-aged adults

**DOI:** 10.18632/aging.204335

**Published:** 2022-10-14

**Authors:** Yu-Tsung Chou, Hung-Yu Chen, I-Hsuan Wu, Fei-Lin Su, Wen-Huang Li, Hung-Lung Hsu, Jui-Ting Tai, Ting-Hsing Chao

**Affiliations:** 1 Department of Family Medicine, National Cheng Kung University Hospital, College of Medicine, National Cheng Kung University, Tainan, Taiwan; 2 Department of Health Management Center, National Cheng Kung University Hospital, College of Medicine, National Cheng Kung University, Tainan, Taiwan; 3 Division of Cardiology, Department of Internal Medicine, National Cheng Kung University Hospital, College of Medicine, National Cheng Kung University, Tainan, Taiwan; 4 Division of Gastroenterology, Department of Internal Medicine, National Cheng Kung University Hospital, College of Medicine, National Cheng Kung University, Tainan, Taiwan

**Keywords:** arterial stiffness, brachial-ankle pulse wave velocity, platelet count, mean platelet volume, middle-aged adult

## Abstract

Background: Platelet counts and mean platelet volume (MPV) are related to cardiovascular disease, but a thorough investigation into the connection between increased arterial stiffness, MPV, and platelet counts is lacking. This study aimed to explore the association of platelet count and MPV with arterial stiffness in young and middle-aged adults.

Methods: A total of 2464 participants who underwent health checkups at National Cheng Kung University Hospital, Taiwan from November 2018 to December 2019 were included. We excluded participants aged <18 or >50 years; who are pregnant; on medication for dyslipidemia; with abnormal platelet count, incomplete data, and past history of hematologic disorders. We examined the association of platelet counts and MPV values with brachial-ankle pulse wave velocity (baPWV) levels and increased arterial stiffness.

Results: Platelet count was significantly higher in participants with increased arterial stiffness than in those without. The multiple linear regression model revealed that platelet counts were positively associated with baPWV levels (β = 1.88, 95% confidence interval (CI): 0.96 to 2.80). In the binary logistic regression analysis, subjects in the higher platelet counts quartiles had a higher risk of developing increased arterial stiffness (Q2 vs. Q1: odds ratio (OR): 1.54, 95% CI: 1.05 to 2.27; Q3 vs. Q1: OR: 1.57, 95% CI: 1.06 to 2.33; and Q4 vs. Q1: OR: 2.23, 95% CI: 1.50 to 3.30). In contrast, MPV levels were not associated with arterial stiffness.

Conclusions: Platelet count in midlife was positively associated with baPWV levels. Participants in higher platelet quartiles were at risk for increased arterial stiffness.

## INTRODUCTION

Arterial stiffness indicates an impaired capability of arterial vessels to expand and contract as a reaction to blood pressure changes [1]. Previous studies have shown that atherosclerotic risk factors such as aging, cigarette smoking, hypertension, diabetes, dyslipidemia, and hyperuricemia are linked to increased arterial stiffness [2, 3]. Furthermore, arterial stiffness is a surrogate marker of atherosclerosis and a risk factor for several cardiovascular (CV) diseases, including stroke [1, 4, 5]. Furthermore, a previous study demonstrated that increased arterial stiffness is associated with both CV disease-associated mortality and all-cause mortality [6]. Therefore, identifying potential risk factors of arterial stiffness may be clinically significant for preventing and managing CV diseases.

In clinical practice, arterial stiffness is commonly assessed by measuring pulse wave velocity (PWV) [7]. Brachial-ankle PWV (baPWV) is measured using a noninvasive vascular screening device evaluating the participant’s blood pressure and pulse waves in the bilateral brachial and tibial arteries. The baPWV value is then calculated by dividing the distance traveled by the pulse wave by the time interval taken for the waveform to travel to such a distance [7]. BaPWV is a well-established index for evaluating arterial stiffness and has been widely used clinically [7–9]. Studies have also demonstrated a positive association between high baPWV value and CV diseases, such as acute myocardial infarction [10], congestive heart failure [11], and even all-cause mortality [12].

Platelets originally played an essential role in the regulation of hemostasis and thrombosis [13]. Furthermore, platelet function and activation were further found to be associated with inflammation and the pathogenesis of atherosclerosis. A high platelet count predicts the risk of acute coronary syndrome [14]. Additionally, mean platelet volume (MPV), one indicator of platelet activation [15], was associated with CV disease [16], hypertension [17], stroke [18], and congestive heart failure [19]. However, studies focusing on arterial stiffness and platelet indicators are very rare. MPV was demonstrated to be associated with baPWV [20, 21]. As for platelet count and arterial stiffness, only one cohort study showed that increased platelet count is related to higher baPWV values in elderly patients with diabetes [22]. However, although platelet counts were shown to be attenuated in the elderly [23] and MPV values may be easily affected by cigarette smoking [24], those confounders were not well considered in previous studies. Furthermore, studies evaluating platelet count in young and middle-aged adults are lacking.

Therefore, we aimed to investigate the association between platelet count and MPV with arterial stiffness in a middle-aged population.

## METHODS

### Study population

**Figure 1:**
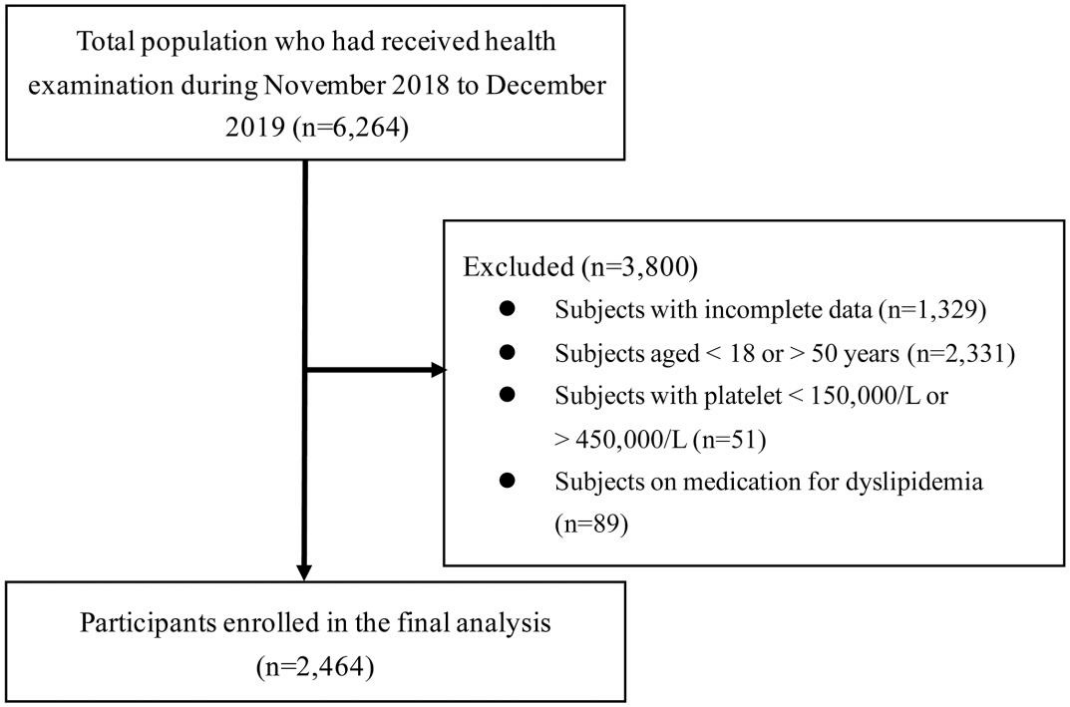
Flow diagram showing the exclusion process for selecting eligible participants.

The participants of this study were recruited from those who underwent health checkups at the National Cheng Kung University Hospital (NCKUH) health management center from November 2018 to December 2019. We excluded subjects aged <18 or >50 years; who are pregnant; on medication for dyslipidemia; and with abnormal platelet count (<150,000/μL or > 450,000/μL), incomplete data, and past history of hematologic disorders. The detailed exclusion process was shown in Figure 1. The analysis was based on delinked secondary data without personally identifiable information, and the institutional review board approved the study protocol at NCKUH (IRB Number: B-ER-108-326). Informed consent was waived because the analysis was based on anonymous data.

All the participants were asked to complete a self-administered questionnaire to assess personal and family medical history and lifestyle factors. The status of cigarette smoking and alcohol use was also obtained, and the participants were categorized into noncurrent and current users. Participants who had smoked at least one pack per month or had alcohol consumption of at least one drink per week for the past 6 months were defined as current smokers and current alcohol users, respectively [25]. Additionally, regular exercisers were individuals who engaged in an intense activity for a minimum of 30 min each time and at least three times per week and were defined to have regular exercise habits.

We measured each subject’s body weight and height, and the body mass index (BMI) was calculated as weight (kg)/height (m^2^). Those with BMI ≥27 kg/m^2^ were obese according to the domestic authority [26]. The right brachial systolic blood pressure (SBP) and diastolic blood pressure (DBP) were measured after the participants rested for at least 10 min. Hypertension was defined as (1) SBP ≥140 mmHg, (2) DBP ≥90 mmHg, or (3) past medical history of hypertension [27]. Then, each participant had their blood drawn to gather biochemical and hematological information. Platelet, white blood cell (WBC), and red blood cell (RBC) counts (WBC) and MPV, hemoglobin, fasting plasma glucose (FPG), glycated hemoglobin (HbA1c), alanine aminotransferase, aspartate aminotransferase, creatinine, total cholesterol (TC), low-density lipoprotein-cholesterol (LDL-C), triglyceride (TG), high-density lipoprotein-cholesterol (HDL-C), high sensitivity C-reactive protein (hs-CRP), and uric acid levels were measured. Diabetes mellitus was defined as (1) FPG level ≥126 mg/dL, (2) HbA1c level ≥6.5%, or (3) a history of diabetes [28]. Hyperuricemia was defined as a serum uric acid level >7.0 mg/dL in men and >6.0 mg/dL in women [29].

### Assessment of platelets

Subjects were further categorized into subgroups based on their platelet count and MPV levels. According to the quartiles of platelet count, we categorized the participants into four subgroups: (1) Q1, platelet count ≥150,000–215,000/μL; (2) Q2, platelet count ≥216,000–248,000/μL; (3) Q3, platelet count ≥249,000–289,000/μL; and (4) Q4, platelet count ≥290,000–450,000/μL. We also divided the participants into quartiles by their MPV levels: (1) Q1, MPV ≥6.2–7.7 fL; (2) Q2, MPV ≥7.8–8.2 fL; (3) Q3, MPV ≥8.3–8.8 fL; and (4) Q4, MPV ≥8.9–11.8 fL.

### Assessment of arterial stiffness

BaPWV was used to evaluate arterial stiffness. The baPWV value was measured using a noninvasive vascular screening device (BP-203RPE II; Colin Medical Technology, Komaki, Japan) with pneumatic pressure cuffs covering the bilateral ankles and upper arms. Each participant’s blood pressure and pulse waves in the bilateral brachial and tibial arteries were simultaneously assessed while in a supine position for at least 5 min. The baPWV value was then calculated automatically by dividing the distance traveled by pulse wave (the distance from the brachial area to the ankle) by the time interval taken for the waveform to travel to such a distance. BaPWV >1400 cm/s were defined as increased arterial stiffness.

### Statistical analysis

SPSS software (v.17.0, SPSS, Inc., Chicago, IL) was used for data analysis. Continuous variables were expressed as mean ± standard deviations, and categorical variables were presented as numbers (percentages). Independent *t*-tests and Pearson’s chi-square analysis were performed both in total subjects and by gender to compare continuous and categorical variables between the participants with and without increased arterial stiffness. In multivariate analysis, both linear and binary logistic regression models were performed to evaluate the association of platelet count and MPV levels with baPWV values and increased arterial stiffness (baPWV >1400 cm/s). Among the adjustment variables were age, sex, BMI, SBP, TC/HDL-C ratio, uric acid, creatinine, hs-CRP, cigarette smoking, and regular exercise. The binary logistic regression model was also conducted by males and females separately to examine the gender difference between the association of platelet-associated parameter and arterial stiffness. A *P* value <0.05 was defined as statistically significant.

## RESULTS

Table 1 compares the demographic characteristics of subjects with and without increased arterial stiffness. Participants with increased arterial stiffness were mostly men and had higher BMI, SBP, DBP, FPG, TC, LDL-C, TG, uric acid, creatinine, WBC, RBC, hemoglobin, and hs-CRP levels than those with normal baPWV levels. Additionally, individuals with increased arterial stiffness had higher hypertension, diabetes, and cigarette smoking rates in total participants. Besides, in both males and females, those with increased arterial stiffness had higher BMI, SBP, DBP, FPG, TC, LDL-C, TG, uric acid, and hs-CRP levels than those with normal baPWV when analyzed separately (shown in Supplementary Table 1).

**Table 1:** Comparisons of clinical characteristics among subjects with and without increased arterial stiffness (baPWV >1,400 cm/s).

**Variables**	**Increased arterial stiffness**		***P* value**
	**No (*n* = 2,125)**	**Yes (*n* = 339)**	
Age, years	40.1 ± 6.5	43.7 ± 4.9	<0.001
Male	1190 (56.0)	275 (81.1)	<0.001
Hypertension	125 (5.9)	144 (42.5)	<0.001
Diabetes mellitus	46 (2.2)	22 (6.5)	<0.001
Hyperuricemia	590 (27.8)	148 (43.7)	<0.001
Current alcohol use	318 (15.0)	85 (25.1)	<0.001
Current smoking	260 (12.2)	71 (20.9)	<0.001
Exercise ≥ 3/wk	935 (44.0)	157 (46.3)	0.426
BMI, kg/m^2^	23.6 ± 3.7	25.4 ± 3.6	<0.001
SBP, mmHg	114.5 ± 11.9	134.8 ± 13.5	<0.001
DBP, mmHg	68.1 ± 9.5	84.1 ± 10.5	<0.001
FPG, mg/dL	91.3 ± 13.8	98.9 ± 28.9	<0.001
ALT, U/L	29.2 ± 27.3	38.9 ± 27.5	<0.001
AST, U/L	24.5 ± 15.0	27.7 ± 12.2	<0.001
Cholesterol, mg/dL	186.1 ± 33.6	198.3 ± 36.0	<0.001
Triglyceride, mg/dL	114.2 ± 75.5	150.8 ± 88.9	<0.001
HDL-C, mg/dL	54.8 ± 15.5	48.7 ± 13.9	<0.001
LDL-C, mg/dL	129.2 ± 33.5	142.0 ± 35.1	<0.001
Cholesterol/HDL-C	3.7 ± 1.2	4.3 ± 1.3	<0.001
Creatinine, mg/dL	0.74 ± 0.18	0.80 ± 0.17	<0.001
Uric acid, mg/dL	5.9 ± 1.5	6.7 ± 1.5	<0.001
WBC, 10^3^/μL	5.8 ± 1.5	6.2 ± 1.6	<0.001
RBC, 10^6^/μL	4.86 ± 0.54	5.13 ± 0.47	<0.001
Hemoglobin, g/dL	14.2 ± 1.6	15.1 ± 1.6	<0.001
hs-CRP, mg/L	1.89 ± 3.59	2.74 ± 4.25	<0.001

Data expressed as mean ± standard deviation or number (percent). Abbreviations: baPWV: brachial-ankle pulse wave velocity; ALT: alanine aminotransferase; AST: aspartate aminotransferase; BMI: body mass index; DBP: diastolic blood pressure; FPG: fasting plasma glucose; HDL-C: high-density lipoprotein-cholesterol; hs-CRP: high sensitivity C-reactive protein; LDL-C: low-density lipoprotein-cholesterol; RBC: red blood cell; SBP: systolic blood pressure; WBC: white blood cell.

**Figure 2:**
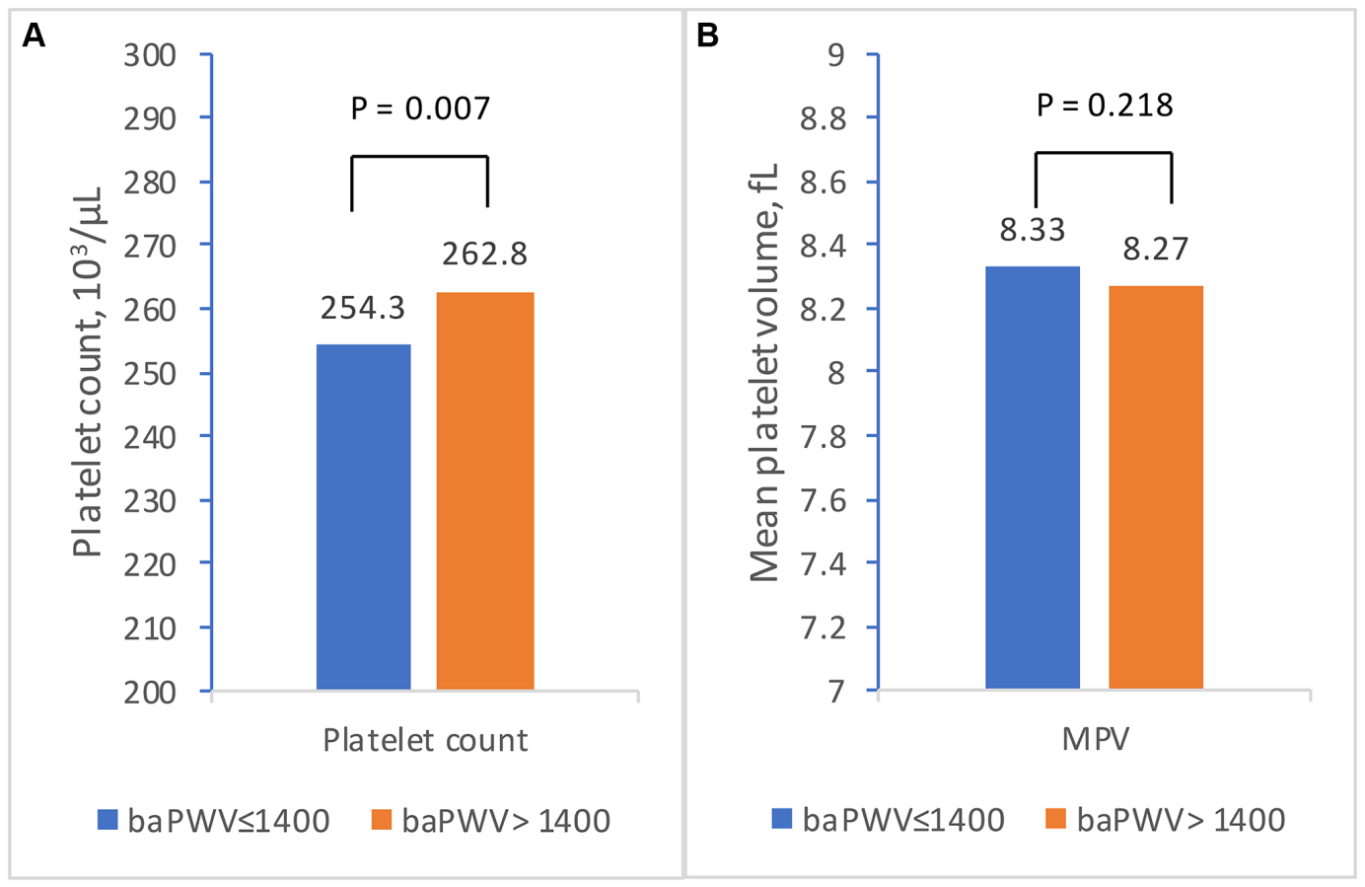
**The relationship between platelet-associated parameters and increased arterial stiffness (baPWV >1,400 cm/s) by independent *t*-test.** (**A**) Comparisons of platelet count between subjects with and without increased arterial stiffness. (**B**) Comparisons of mean platelet volume subjects with and without increased arterial stiffness. The orange and blue bars represent the mean levels in subjects with and without increased arterial stiffness, respectively. A *P* value < 0.05 was defined as statistically significant. Abbreviations: baPWV: brachial-ankle pulse wave velocity; MPV: mean platelet volume.

Figure 2 shows the comparisons between platelet-associated parameters and increased arterial stiffness in total participants by univariate analysis. The results showed that platelet count was significantly higher in subjects with increased arterial stiffness than in those without (platelet count: 254.3 ± 53.8 × 10^3^/μL vs. 262.8 ± 52.4 × 10^3^/μL, *P* = 0.007). Additionally, Table 2 demonstrated that higher platelet count quartiles exhibited significantly higher prevalence of arterial stiffness than lower platelet count quartiles. Contrarily, there was no statistical difference in MPV levels among subjects with or without arterial stiffness. When examined individually by gender, the platelet counts were significantly higher in those with increased arterial stiffness in both males and females, whereas there was no significant difference of MPV values in those with and without increased arterial stiffness (shown in Supplementary Figures 1 and 2). While performing the linear regression analysis (shown in Table 3), we initially investigated the association of baPWV value with platelet count and MPV separately. The results showed that platelet count was positively associated with baPWV values (β = 1.82, 95% confidence interval (CI): 0.51 to 3.13, *P* = 0.007), whereas there was an inverse relationship between MPV and baPWV values (β = −11.85, 95% CI: −20.36 to −3.34, *P* = 0.006). The relationship between increased arterial stiffness, platelet count, and MPV level was further examined using a multiple linear regression model. The results showed that platelet count remained positively associated with baPWV after adjusting potential confounders (β = 1.88, 95% CI: 0.96 to 2.80, *P* = 0.007). However, the relationship between MPV and baPWV levels became insignificant after multivariable adjustment (β = −2.68, 95% CI: −8.39 to 3.03, *P* = 0.358). Age; male sex; BMI; blood pressure; blood glucose, TC, uric acid, and hs-CRP levels; and cigarette smoking were also independently and positively correlated with baPWV value. Simultaneously, exercise habit was negatively related to baPWV value.

**Table 2:** Univariate analysis of the relationship between platelet-associated parameters and increased arterial stiffness (baPWV >1,400 cm/s).

**Variables**	**Increased arterial stiffness**		***P* value**
	**No (*n* = 2,125)**	**Yes (*n* = 339)**	
Platelet count, 10^3^/μL	254.3±53.8	262.8±52.4	0.007
Platelet count, quartiles			0.023
Q1 (150~215, 10^3^/μL)	555 (26.1)	64 (18.9)	
Q2 (216~248, 10^3^/μL)	518 (24.4)	89 (26.3)	
Q3 (249~289, 10^3^/μL)	*533 (25.1)*	86 (25.4)	
Q4 (290~450, 10^3^/μL)	519 (24.4)	100 (29.5)	
**Mean platelet volume, fL**	8.33±0.83	8.27±0.80	0.218
**Mean platelet volume, quartiles**			0.499
Q1 (6.2~7.7 fL)	526 (24.8)	97 (28.6)	
Q2 (7.8~ 8.2 fL)	528 (24.8)	79 (23.3)	
Q3 (8.3~ 8.8 fL)	536 (25.2)	80 (23.6)	
Q4 (8.9~11.8 fL)	535 (25.2)	83 (24.5)	

Data expressed as mean ± standard deviation or number (percent). Abbreviation: baPWV: brachial-ankle pulse wave velocity.

**Table 3:** Linear regression model for baPWV value (cm/s) with platelet count and mean platelet volume levels.

**Variables**	**Univariate**				**Multivariate**	
	**β (95% CI)**	***P* value**	**β (95% CI)**	***P* value**	**β (95% CI)**	***P* value**
Platelet count, 10^4^/μL	1.82 (0.51~3.13)	0.007	1.88 (0.96~2.80)	<0.001		
Mean platelet volume, fL	−11.85(−20.36~−3.34)	0.006			−2.68(-8.39~−3.03)	0.358
Age, years			4.93 (4.17~5.70)	<0.001	4.86 (4.09~5.62)	<0.001
Sex, male			28.90 (12.25~−45.56)	0.001	22.32 (5.87~−38.78)	0.008
BMI, kg/m^2^			−9.11 (−10.71~−7.51)	<0.001	−9.04 (−10.64~−7.44)	<0.001
SBP, mmHg			8.46 (8.05~8.87)	<0.001	8.53 (8.12~8.94)	<0.001
FPG, mg/dL			0.50 (0.21~0.80)	0.001	0.50 (0.20~0.80)	0.001
Cholesterol/HDL-C ratio			9.11 (4.28~13.94)	<0.001	10.30 (5.50~15.11)	<0.001
Uric acid, mg/dL			6.44 (2.19~10.68)	0.003	6.66 (2.40~10.91)	0.002
Creatinine, mg/dL			−29.68 (−70.52~11.16)	0.154	−32.34 (−73.29~8.62)	0.122
hs-CRP, mg/L			2.01 (0.67~3.35)	0.003	2.13 (0.79~3.47)	0.002
Smoking, yes vs. no			14.55 (0.22~28.88)	0.047	14.68 (0.30~29.05)	0.045
Exercise, yes vs. no			−9.10 (−18.08~0.61)	0.066	−10.10 (−19.38~−0.38)	0.042

Abbreviations: baPWV: brachial-ankle pulse wave velocity; BMI: body mass index; CI: confidence interval; FPG: fasting plasma glucose; HDL-C: high-density lipoprotein-cholesterol; hs-CRP: high sensitivity C-reactive protein; SBP: systolic blood pressure.

The risk of increased arterial stiffness among subjects with different levels of platelet count and MPV level was further analyzed using the binary logistic regression model (Table 4). The results revealed that, compared with subjects in the lowest quartile (Q1), those in high quartiles (Q2–Q4) were all found to have a higher risk of increased arterial stiffness (Q2 vs. Q1: odds ratio (OR):1.54, 95% CI: 1.05 to 2.27, *P* = 0.029; Q3 vs. Q1: OR: 1.57, 95% CI: 1.06 to 2.33, *P* = 0.026; Q4 vs. Q1: OR: 2.23, 95% CI: 1.50 to 3.30, *P* < 0.001) after adjusting for age, sex, obesity, diabetes, hypertension, dyslipidemia, hyperuricemia, hs-CRP level, cigarette smoking, and regular exercise. In addition, the positive relationship between platelet and increased arterial stiffness remained significant when the logistic regression model was conducted by gender (shown in Supplementary Tables 2 and 3). Contrarily, there was no statistically significant difference in the risk of increased arterial stiffness among patients across different MPV quartiles in total (shown in Table 4), male (shown in Supplementary Table 2) and female participants (shown in Supplementary Table 3).

**Table 4:** Logistic regression model for increased arterial stiffness (baPWV >1,400 cm/s) with platelet count and mean platelet volume levels.

**Variables**	**Crude OR (95% CI)**	***P* value**	**Adjusted OR^a^ (95% CI)**	***P* value**
**Platelet count, quartiles**				
Q1 (150~215, 10^3^/μL)	Reference		Reference	
Q2 (216~248, 10^3^/μL)	1.49 (1.06–2.10)	0.023	1.54 (1.05–2.27)	0.029
Q3 (249~289, 10^3^/μL)	1.38 (0.98–1.95)	0.067	1.57 (1.06–2.33)	0.026
Q4 (290~450, 10^3^/μL)	1.61 (1.15–2.26)	0.006	2.23 (1.50–3.30)	<0.001
**Mean platelet volume, quartiles**				
Q1 (6.2~7.7 fL)	Reference		Reference	
Q2 (7.8~8.2 fL)	0.84 (0.61–1.16)	0.296	0.96 (0.67–1.38)	0.827
Q3 (8.3~8.8 fL)	0.83 (0.60–1.14)	0.251	0.99 (0.69–1.42)	0.940
Q4 (8.9~11.8 fL)	0.87 (0.64–1.20)	0.404	0.86 (0.60–1.24)	0.860

Abbreviations: baPWV: brachial-ankle pulse wave velocity; CI: confidence interval; OR: odds ratio. ^a^adjusted for age, sex, obesity, hypertension, diabetes, hyperuricemia, total cholesterol/high-density lipoprotein-cholesterol ratio, high sensitivity C-reactive protein, cigarette smoking and regular exercise.

## DISCUSSION

### Principal findings

This is the first study focusing on the association of platelet count and arterial stiffness in young and middle-aged populations with total adjustment of traditional risk factors of arterial stiffness, such as age; obesity; smoking; blood pressure; or FPG, uric acid, hs-CRP, or lipid profile levels. According to our research, young and middle-aged adults are significantly associated with a high risk of increased arterial stiffness when their platelet counts are elevated, even within the normal range.

### Platelet-associated indices and arterial stiffness in previous studies

#### Platelet count and arterial stiffness

According to previous studies, platelet disorders with abnormally high and low platelet count, such as essential thrombocythemia and idiopathic thrombocytopenia purpura, were related to the risk of increased arterial stiffness [14, 30–32]. Although platelet activity is associated with CV diseases [16, 33] and the process of atherogenesis [34], studies focusing on platelet-associated parameters such as platelet count and MPV with arterial stiffness in general population remain extremely limited [20–22]. Only two studies discussed the correlation between arterial stiffness and platelet count [20, 22]. One study from Liu et al. found that increased platelet count was positively related to baPWV value in subjects with diabetes but not those without [22] and the other study from Marina et al. showed insignificant relationship between platelet count and arterial stiffness [20]. However, the population was relatively old in both studies from Liu et al. (mean age: 66.8 years for those without diabetes) and Mariana et al. (mean age: 55.3 years and 54.7 years in males and females). Because elevated platelet counts were correlated with increased mortality [35, 36], evaluation of platelet count and arterial stiffness in elderly adults may be potentially interfered with survivorship bias. Besides, the platelet count is relatively stable in young adulthood and midlife, and then starts to decline in people’s fifties and sixties [23, 37, 38]. Considering that platelet count is attenuated in the elderly [23, 37, 38], our study had the advantage of minimizing the confounding effect of age in the relationship between platelet count and arterial stiffness by taking aim at the young and middle-aged population. Additionally, although studies showed gender difference between both arterial stiffness [39] and platelet count [40], these significant disparities were not considered in the two aforementioned studies [20, 22]. Furthermore, the arterial stiffness was evaluated using the augmentation index [20] in study from Marina et al., which have several limitations in the assessment of wave reflection [41].

#### MPV and arterial stiffness

Two cross-sectional studies from Wang et al. and Marina et al. showed a positive relationship between MPV and increased arterial stiffness [20, 21] which was different from the findings of this study. However, despite that hematologic malignancy and abnormal platelet count (including thrombocytopenia and thrombocytosis) were found to affect the pathogenesis of arterial stiffness [30, 42, 43], subjects with hematologic disorders or abnormal platelet counts were not excluded from those two studies [20, 21]. Besides, several factors such as hyperuricemia and exercise habit might play a role in the pathogenesis of arterial stiffness, but these parameters were not adjusted in the regression model. In addition, the population in the study from Wang et al. had relatively high prevalence of cigarette smoking (approximately 37.5%); however, the habit of cigarette smoking was excluded in the final regression analysis. Considering that cigarette smoking leads to increased arterial stiffness [44] and also significantly elevates the MPV value [24], excluding cigarette smoking in the regression analysis may confound the final results [44]. Consequently, the disparity between our results and those of earlier studies [20–22] may be due to several unadjusted confounding factors and different population characteristics that were fully explored in the current study.

### Possible mechanism of platelet counts and increased arterial stiffness

It is well recognized that extracellular matrix, inflammatory molecules, endothelial cell dysfunction, and oxidative stress interact to cause increased arterial stiffness [5, 45]. A previous study had found that platelets release several matrix metalloproteinases (MMP), such as MMP-1, MMP-2, MMP-3, MMP-9, and MMP-14 [46]. An *in vitro* study also revealed a positive association between platelet count and MMP level [47]. Considering that MMP-2, MMP-3, and MMP-9 are directly involved in the pathogenesis of arterial stiffness by degradation of elastic fiber with resultant reduced arterial elasticity [48–50], it is reasonable to hypothesize that subjects with high platelet count, potentially with higher MMP levels, may have increased risk of arterial stiffness. In addition, platelets contain granules that may cause elevation of chemokines or inflammatory cytokines such as transforming growth factor-beta [51] and interleukin-1 [52], which were all found to be associated with arterial stiffness [53, 54]. Furthermore, studies have demonstrated that platelet count was positively related to the production of transforming growth factor-beta and interleukin-1 [55–58], which might also result in the progression of arterial stiffness.

Another possible explanation for platelet count and arterial stiffness may be its role in the CD40–CD40 ligand interaction. CD40 and CD40 ligands are well known for involving vascular and systemic inflammation and the pathogenesis of CV diseases [59, 60]. The CD40 ligands were expressed by nonhematopoietic and hematopoietic cells, and the soluble form of CD40 ligands in the circulation was mainly generated from platelets [61]. In a previous study, platelet counts correlated highly with a soluble form of CD40 ligand concentrations [62]. Because platelets also expressed CD40, an elevated platelet count may represent a high activity of CD40–CD40 ligand signaling and thus contribute to vascular inflammation, leading to increased arterial stiffness.

### Limitations

Although our study had the advantage of a relatively large sample size with comprehensive personal and medical information, including past history, lifestyle habits, and laboratory results, some limitations should be addressed. First, because of the cross-sectional design, it is difficult to establish a causal relationship between platelet-associated parameters and arterial stiffness. Second, because our analysis was confined to a Taiwanese population, whether it is plausible to extrapolate these results to other ethnic groups may need further investigation. Third, our participants were recruited from the health examination center of a tertiary medical center with potential selection bias. Fourth, although MMPs, transforming growth factor-beta, and interleukin-1 were associated with platelet count and arterial stiffness, these data were unavailable in the current study. Further investigation with more thorough information about such crucial biomarkers might be necessary for verifying the relationship and possible pathogenesis between platelet count and arterial stiffness.

In conclusion, the platelet counts in young age and midlife, even within normal range were positively associated with baPWV levels without gender difference. Additionally, participants in high platelet count quartiles (Q2–Q4) were related to an increased risk of arterial stiffness. In contrast, the relationship between MPV levels and arterial stiffness was insignificant. Our data suggest that platelet count is a useful marker of arterial stiffness in young and middle-aged adults. A high-normal platelet count should prompt further evaluation of potential atherosclerosis. The clinical application of this widely available marker in risk stratification warrants further investigation.

## Supplementary Materials

Supplementary Figures

Supplementary Tables
