# Supplementary material for: Higher platelet count, even within normal range, is associated with increased arterial stiffness in young and middle-aged adults
Source: Aging (Albany NY). 2022 Oct 14;14(19):8061–76. doi: 10.18632/aging.204335 (PMC9596195; doi:10.18632/aging.204335)
Supplement: Supplementary Figures [file aging-14-204335-s001.pdf]

SUPPLEMENTARY FIGURES

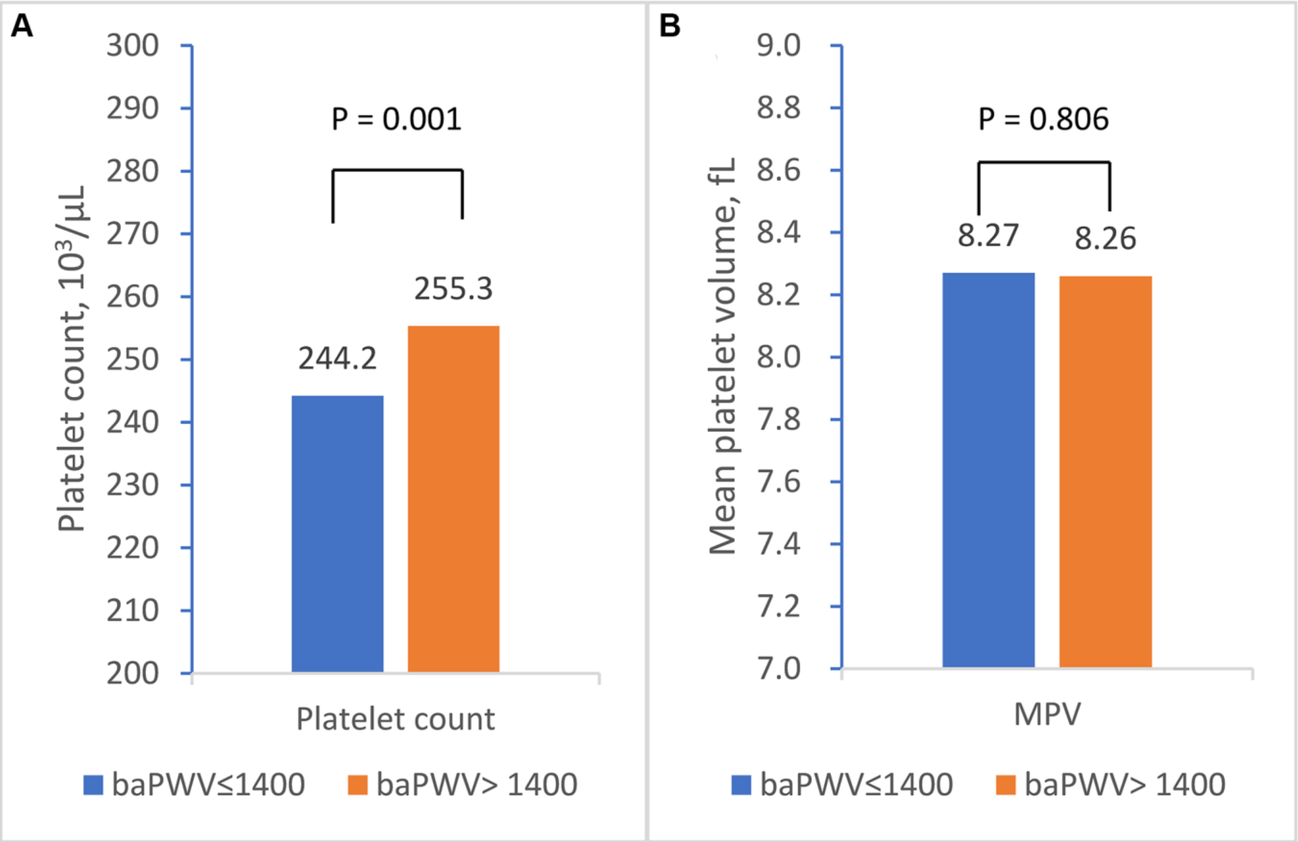

Supplementary Figure 1. The relationship between platelet-associated parameters and increased arterial stiffness (baPWV >1,400 cm/s) in males by independent *t*-test. (**A**) Comparisons of platelet count between subjects with and without increased arterial stiffness. (**B**) Comparisons of mean platelet volume subjects with and without increased arterial stiffness. The orange and blue bars represent the mean levels in subjects with and without increased arterial stiffness, respectively. A *P* value < 0.05 was defined as statistically significant. Abbreviations: baPWV: brachial-ankle pulse wave velocity; MPV: mean platelet volume.

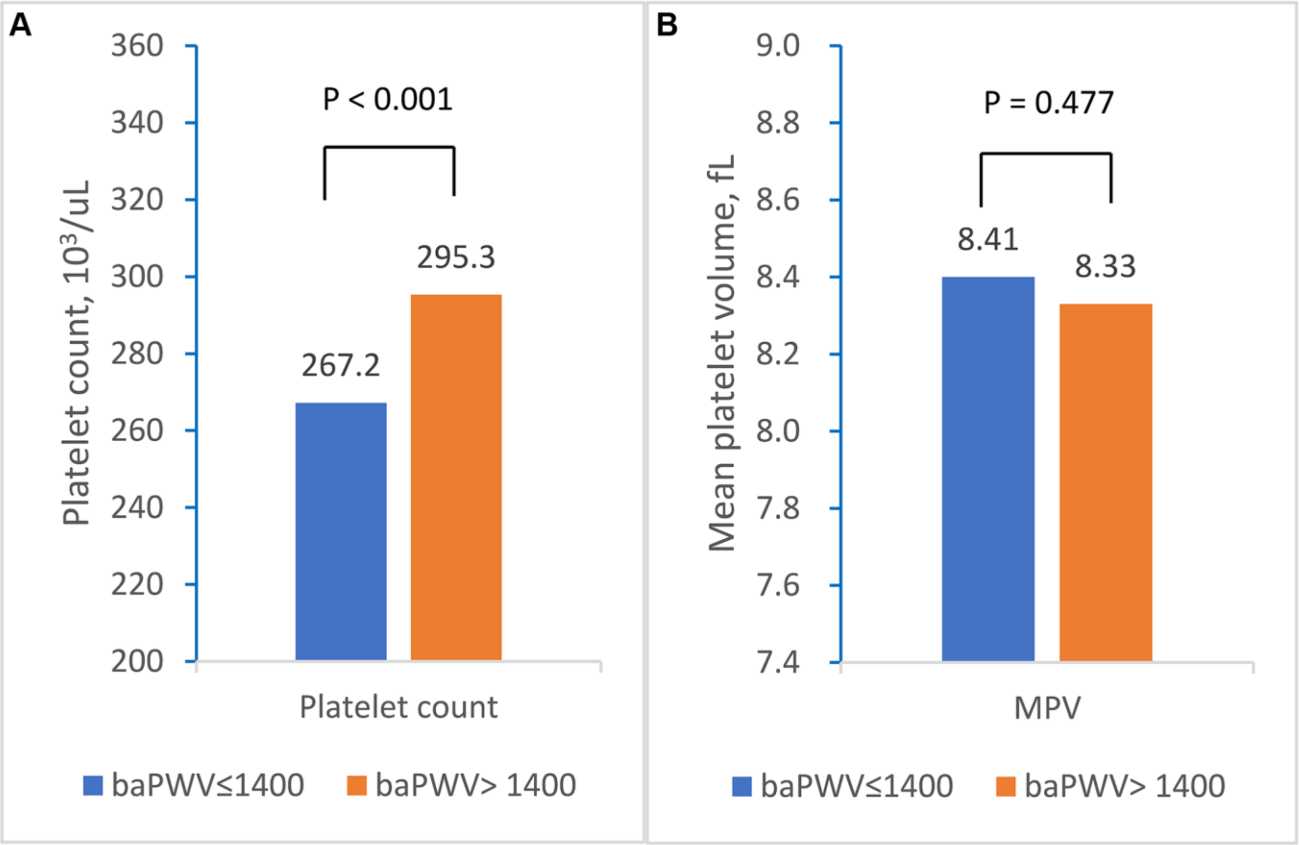

Supplementary Figure 2. The relationship between platelet-associated parameters and increased arterial stiffness (baPWV >1,400 cm/s) in females by independent *t*-test. (**A**) Comparisons of platelet count between subjects with and without increased arterial stiffness. (**B**) Comparisons of mean platelet volume subjects with and without increased arterial stiffness. The orange and blue bars represent the mean levels in subjects with and without increased arterial stiffness, respectively. A *P* value < 0.05 was defined as statistically significant. Abbreviations: baPWV: brachial-ankle pulse wave velocity; MPV: mean platelet volume.
