# Supplementary material for: Higher platelet count, even within normal range, is associated with increased arterial stiffness in young and middle-aged adults
Source: Aging (Albany NY). 2022 Oct 14;14(19):8061–76. doi: 10.18632/aging.204335 (PMC9596195; doi:10.18632/aging.204335)
Supplement: Supplementary Tables [file aging-14-204335-s002.pdf]

SUPPLEMENTARY TABLES

Supplementary Table 1. Comparisons of clinical characteristics among subjects with and without increased arterial stiffness (baPWV >1,400 cm/s) in males and females.

Variables	Male		*P* value	Female		*P* value
	Increased arterial stiffness			Increased arterial stiffness		
	No (*n* = 1,190)	Yes (*n* = 275)		No (*n* = 935)	Yes (*n* = 64)	
Age, years	40.6 ± 6.3	43.5 ± 4.9	<0.001	39.5 ± 6.7	44.8 ± 4.5	<0.001
Hypertension	103 (8.7)	118 (42.9)	<0.001	22 (2.4)	26 (40.6)	<0.001
Diabetes mellitus	31 (2.6)	20 (7.3)	<0.001	15 (1.6)	2 (3.1)	0.363
Hyperuricemia	477 (40.1)	138 (50.2)	0.002	113 (12.2)	10 (15.6)	0.404
Current alcohol use	258 (21.7)	76 (27.6)	0.020	60 (6.4)	9 (14.1)	0.034
Current smoking	232 (19.5)	66 (24.0)	0.094	28 (3.0)	5 (7.8)	0.037
Exercise ≥ 3/wk	564 (47.4)	126 (45.8)	0.637	371 (39.7)	31 (48.4)	0.167
BMI, kg/m^2	24.7 ± 3.4	25.8 ± 3.4	<0.001	22.1 ± 3.6	23.7 ± 4.1	0.001
SBP, mmHg	119.5 ± 10.5	134.6 ± 13.3	<0.001	108.2 ± 10.6	135.8 ± 14.4	<0.001
DBP, mmHg	72.0 ± 8.5	84.8 ± 10.2	<0.001	63.2 ± 8.2	81.3 ± 11.4	<0.001
FPG, mg/dL	93.6 ± 15.1	99.9 ± 31.5	0.001	88.4 ± 11.4	94.5 ± 11.5	<0.001
ALT, U/L	35.2 ± 24.1	42.9 ± 28.5	<0.001	21.4 ± 29.1	21.8 ± 12.3	0.907
AST, U/L	26.5 ± 12.7	29.1 ± 12.7	0.003	21.4 ± 17.1	21.8 ± 7.4	0.996
Cholesterol, mg/dL	189.8 ± 33.7	198.7 ± 35.9	<0.001	181.4 ± 32.9	196.7 ± 36.8	<0.001
Triglyceride, mg/dL	135.1 ± 86.1	159.9 ± 91.7	<0.001	87.6 ± 47.5	111.8 ± 62.8	0.003
HDL-C, mg/dL	48.8 ± 12.5	46.9 ± 13.2	0.027	62.5 ± 15.5	56.6 ± 14.3	0.004
LDL-C, mg/dL	135.7 ± 33.1	142.8 ± 35.3	0.002	120.8 ± 32.1	138.8 ± 34.4	<0.001
Cholesterol/HDL-C	4.1 ± 1.2	4.5 ± 1.3	<0.001	3.1 ± 0.9	3.6 ± 0.9	<0.001
Creatinine, mg/dL	0.86 ± 0.13	0.85 ± 0.13	0.413	0.58 ± 0.11	0.57 ± 0.12	0.284
Uric acid, mg/dL	6.8 ± 1.3	7.0 ± 1.3	0.005	4.8 ± 1.1	5.3 ± 1.2	0.001
WBC, 10^3/μL	5.8 ± 1.5	6.2 ± 1.6	<0.001	5.7 ± 1.6	5.9 ± 1.5	0.269
RBC, 10^6/μL	5.14 ± 0.45	5.22 ± 0.44	0.005	4.51 ± 0.44	4.71 ± 0.37	<0.001
Hemoglobin, g/dL	15.3 ± 1.0	15.6 ± 1.0	<0.001	12.9 ± 1.3	13.0 ± 1.8	0.679
hs-CRP, mg/L	1.85 ± 2.72	2.67 ± 4.11	0.002	1.93 ± 4.47	3.05 ± 4.86	0.078

Data expressed as mean ± standard deviation or number (percent). Abbreviations: baPWV: brachial-ankle pulse wave velocity; ALT: alanine aminotransferase; AST: aspartate aminotransferase; BMI: body mass index; DBP: diastolic blood pressure; FPG: fasting plasma glucose; HDL-C: high-density lipoprotein-cholesterol; hs-CRP: high sensitivity C-reactive protein; LDL-C: low-density lipoprotein-cholesterol; RBC: red blood cell; SBP: systolic blood pressure; WBC: white blood cell.

Supplementary Table 2. Logistic regression model for increased arterial stiffness (baPWV >1,400 cm/s) with platelet count and mean platelet volume levels in males.

Variables	Crude OR (95% CI)	*P* value	Adjusted OR[a] (95% CI)	*P* value
**Platelet count, quartiles**				
Q1 (150~209, 10^3/μL)	Reference		Reference	
Q2 (210~241, 10^3/μL)	1.33 (0.89–1.99)	0.170	1.42 (0.91–2.22)	0.120
Q3 (242~274, 10^3/μL)	1.68 (1.14–2.48)	0.009	1.61 (1.04–2.48)	0.032
Q4 (275~450, 10^3/μL)	1.65 (1.12–2.44)	0.012	1.76 (1.14–2.73)	0.011
**Mean platelet volume, quartiles**				
Q1 (6.4~7.6 fL)	Reference		Reference	
Q2 (7.7~8.1 fL)	0.78 (0.54–1.15)	0.207	1.32 (0.56–3.11)	0.523
Q3 (8.2~8.7 fL)	0.80 (0.55–1.15)	0.230	1.05 (0.44–2.48)	0.920
Q4 (8.8~11.5 fL)	0.88 (0.61–1.26)	0.477	1.04 (0.43–2.51)	0.933

Abbreviations: baPWV: brachial-ankle pulse wave velocity; CI: confidence interval; OR: odds ratio. [a]adjusted for age, obesity, hypertension, diabetes, hyperuricemia, total cholesterol/high-density lipoprotein-cholesterol ratio, high sensitivity C-reactive protein, cigarette smoking and regular exercise.

Supplementary Table 3. Logistic regression model for increased arterial stiffness (baPWV >1,400 cm/s) with platelet count and mean platelet volume levels in females.

Variables	Crude OR (95% CI)	*P* value	Adjusted OR[a] (95% CI)	*P* value
**Platelet count, quartiles**				
Q1 (152~223, 10^3/μL)	Reference		Reference	
Q2 (224~261, 10^3/μL)	2.07 (0.77–5.53)	0.148	2.37 (0.77–7.30)	0.133
Q3 (262~304, 10^3/μL)	2.44 (0.93–6.39)	0.070	3.31 (1.09–10.01)	0.034
Q4 (306~450, 10^3/μL)	4.85 (1.98–11.92)	0.001	3.94 (1.39–11.12)	0.010
**Mean platelet volume, quartiles**				
Q1 (6.3~7.7 fL)	Reference		Reference	
Q2 (7.8~8.2 fL)	0.84 (0.61–1.16)	0.296	0.80 (0.53–1.22)	0.308
Q3 (8.3~8.8 fL)	0.83 (0.60–1.14)	0.251	0.92 (0.62–1.39)	0.703
Q4 (8.9~11.8 fL)	0.87 (0.64–1.20)	0.404	0.76 (0.51–1.13)	0.177

Abbreviations: baPWV: brachial-ankle pulse wave velocity; CI: confidence interval; OR: odds ratio. [a]adjusted for age, obesity, hypertension, diabetes, hyperuricemia, total cholesterol/high-density lipoprotein-cholesterol ratio, high sensitivity C-reactive protein, cigarette smoking and regular exercise.
